# The potential role of Antarctic krill faecal pellets in efficient carbon export at the marginal ice zone of the South Orkney Islands in spring

**DOI:** 10.1007/s00300-017-2118-z

**Published:** 2017-04-13

**Authors:** A. Belcher, G. A. Tarling, C. Manno, A. Atkinson, P. Ward, G. Skaret, S. Fielding, S. A. Henson, R. Sanders

**Affiliations:** 1 National Oceanography Centre, Southampton, SO14 3ZH UK; 2 University of Southampton, Southampton, SO14 3ZH UK; 3 British Antarctic Survey, Cambridge, CB3 0ET UK; 4 Plymouth Marine Laboratory, Prospect Place, The Hoe, Plymouth, PL1 3DH UK; 5 Institute of Marine Research, PO Box 1870, Nordnes, 5817 Bergen, Norway

**Keywords:** Faecal pellet attenuation, Antarctic krill, Southern Ocean, Carbon flux, Faecal pellet

## Abstract

Antarctic krill (*Euphausia superba*) play a central role in the food web of the Southern Ocean, forming a link between primary production and large predators. Krill produce large, faecal pellets (FP) which can form a large component of mesopelagic particulate organic carbon (POC) fluxes. However, the patchy distribution of krill swarms, highly variable pellet composition, and variable sinking and attenuation rates means that these episodic, but potentially large, carbon fluxes are difficult to sample or model. We measured particle flux and type using Marine Snow Catchers (MSC) in the marginal ice zone near the South Orkneys, Antarctica. Krill FP were the dominant component of the POC flux in the upper 200 m (typically 60–85%). FP sinking velocities measured onboard were highly variable (15–507 m d^−1^) but overall high, with mean equivalent velocities of 172, 267, and 161 m d^−1^ at our three stations. The high numbers of krill FP sinking through the mesopelagic suggest that krill FP can be transferred efficiently and/or that rates of krill FP production are high. We compared our direct MSC-derived estimates of krill FP POC flux (33–154 mg C m^−2^ d^−1^) and attenuation to estimates of krill FP production based on previous measurements of krill density and literature FP egestion rates, and estimated net krill FP attenuation rates in the upper mesopelagic. Calculated attenuation rates are sensitive to krill densities in the overlying water column but suggest that krill FP could be transferred efficiently through the upper mesopelagic, and, in agreement with our MSC attenuation estimates, could make large contributions to bathypelagic POC fluxes. Our study contrasts with some others which suggest rapid FP attenuation, highlighting the need for further work to constrain attenuation rates and assess how important the contribution of Antarctic krill FP could be to the Southern Ocean biological carbon pump.

**Electronic supplementary material:**

The online version of this article (doi:10.1007/s00300-017-2118-z) contains supplementary material, which is available to authorized users.

## Introduction

The Southern Ocean is an important part of the global carbon cycle, with the region south of 40°S (~21% of the ocean area) estimated to account for 26 ± 6% of global export production (defined as the material exported out of the surface ocean) (Primeau et al. 2013). Empirical models suggest that the export of material out of the euphotic zone in the Southern Ocean is efficient but that the transfer of this material through the mesopelagic to the deep ocean is inefficient (Henson et al. 2012). The efficiency with which carbon sinks through the mesopelagic by the biological carbon pump (BCP) is intrinsically linked to levels of atmospheric CO_2_ (Kwon et al. 2009). Furthermore, the BCP in much of the Southern Ocean does not operate at maximum efficiency as primary production is limited by factors including light and iron (Boyd et al. 1999), leading to upwelled nutrients being subducted unused into the ocean interior.

However, this picture conceals the existence of high flux events in some regions of the Southern Ocean, associated with sinking diatoms (e.g., Smetacek 1985; Beaulieu 2002; Roca-Marti et al. 2015), diatom resting spores (Rembauville et al. 2015), enhanced iron supply (e.g., Bidigare et al. 1999; Savoye et al. 2008; Pollard et al. 2009; Smetacek et al. 2012; Jouandet et al. 2014), and the influence of marginal ice zones (Smith and Nelson 1985; Fischer et al. 1988; Buesseler et al. 2001; Cavan et al. 2015). Short-term, high export events can make up a substantial fraction of the annual POC flux. In the Bransfield Strait, 97% of the annual POC flux to 1588 m occurred in the two most productive months, associated with *Euphausia superba* (herein referred to as krill) faecal pellets, (Bodungen et al. 1987; Wefer et al. 1988), yet limited observations in these marginal ice zones mean that krill and their large FP fluxes are not well represented in global biogeochemical models.

Krill are present in high densities in some regions of the Southern Ocean (Atkinson et al. 2008) often occurring in large swarms (Hamner et al. 1989), resulting in the production of large numbers of faecal pellets (FP) some of which can sink rapidly through the water column (Atkinson et al. 2012). Cadée (1992) deployed floating sediment traps for <1 day at 50–75 and 150 m in the Weddell-Scotia Sea, observing that krill FP dominated the trap material. One station in particular (out of 5) exhibited exceptionally high POC fluxes (0.7 g C m^−2^ d^−1^ at 75 m and 1.5 g C m^−2^ d^−1^ at 150 m) dominated by krill FP. Cadée (1992) postulated that this large flux event was related to a krill swarm, observed following trap deployment. High krill FP fluxes have also been observed during short-term sediment trap deployments in the Bransfield Strait and Weddell Sea (Dunbar 1984; Bodungen et al. 1987; Bathmann et al. 1991). Similarly, a long-term sediment trap study in Terra Nova Bay in the Ross Sea noted atypically high POC fluxes (11.13 mg m^−2^ d^−1^ at 95 m) in late April–mid June (Accornero et al. 2003), dominated by cylindrical faecal pellets (74–80% of FP) suggesting that large swarms of euphausiids could also be important contributors to flux in the winter. Observational evidence, therefore, suggests that krill FP can both provide a vehicle for high export of POC, and may also transfer carbon efficiently through the mesopelagic. The South Orkney Islands, situated in the spring–summer marginal ice zone, have been found to exhibit high abundances of krill (Hewitt et al. 2004; Siegel et al. 2004; Atkinson et al. 2008), yet the contribution of their FP to total POC flux there has not been quantified.

Faecal pellets can also be broken up and remineralised by zooplankton and prokaryotes as they sink, decreasing the efficiency of export to the deep sea (e.g., Poulsen and Kiørboe 2005; Iversen and Poulsen 2007; Svensen et al. 2012, 2014) and fragmentation has been suggested to explain high retention of krill FP in the study of González (1992). González (1992) suggests that where krill FP production is high relative to abundances of the zooplankton recycling community, a large percentage of krill FP can pass undisturbed through the mesopelagic zone and reach deep sediment traps. Even if krill FP are grazed upon by deeper dwelling zooplankton populations, this could still result in fresh pellets reaching the deep sea via a cascade effect (Urrere and Knauer 1981; Bodungen et al. 1987; Miquel et al. 2015; Belcher et al. 2017). These uncertainties are compounded by the great variability in sinking rates of the pellets, which depends on diet (Cadée et al. 1992; Atkinson et al. 2012). For instance, the most carbon-rich pellets were found to sink only at an equivalent of a few 10 s of meters per day, similar to sinking rates of copepod pellets (Atkinson et al. 2012). Because faecal pellets are major vectors of iron and other essential nutrients in addition to carbon (Schmidt et al. 2012, 2016), a better understanding of the balance between retention and export of these particles is needed.

Krill occur mostly in swarms which may produce enough FP to overload the capacity of the zooplankton and microbial communities to graze, fragment, and/or remineralise their FP before they sink to the ocean interior (Atkinson et al. 2012). However, the patchy distribution of krill swarms and the highly variable composition and sinking velocities of the FP (Cadée et al. 1992; Atkinson et al. 2012) mean that their contribution to flux can be episodic and, therefore, difficult to sample. In addition, the complex behaviour of krill makes it difficult to replicate natural conditions in the laboratory (Gibbons et al. 1999; Schmidt and Atkinson 2016; Tarling and Fielding 2016). Overall, this potentially large flux of POC to the mesopelagic of the Southern Ocean is poorly quantified and it is not yet clear from previous studies whether krill FP are transferred efficiently through the upper mesopelagic.

We made measurements of particle flux in the marginal ice zone (MIZ) near the South Orkney Islands during austral spring of two seasons. Our aims were first to quantify the relative proportion of krill pellets in the sinking material, second, to estimate carbon fluxes in the upper mesopelagic, and third, to gauge the vertical transfer efficiency.

## Methods

### Study site

**Fig. 1:**
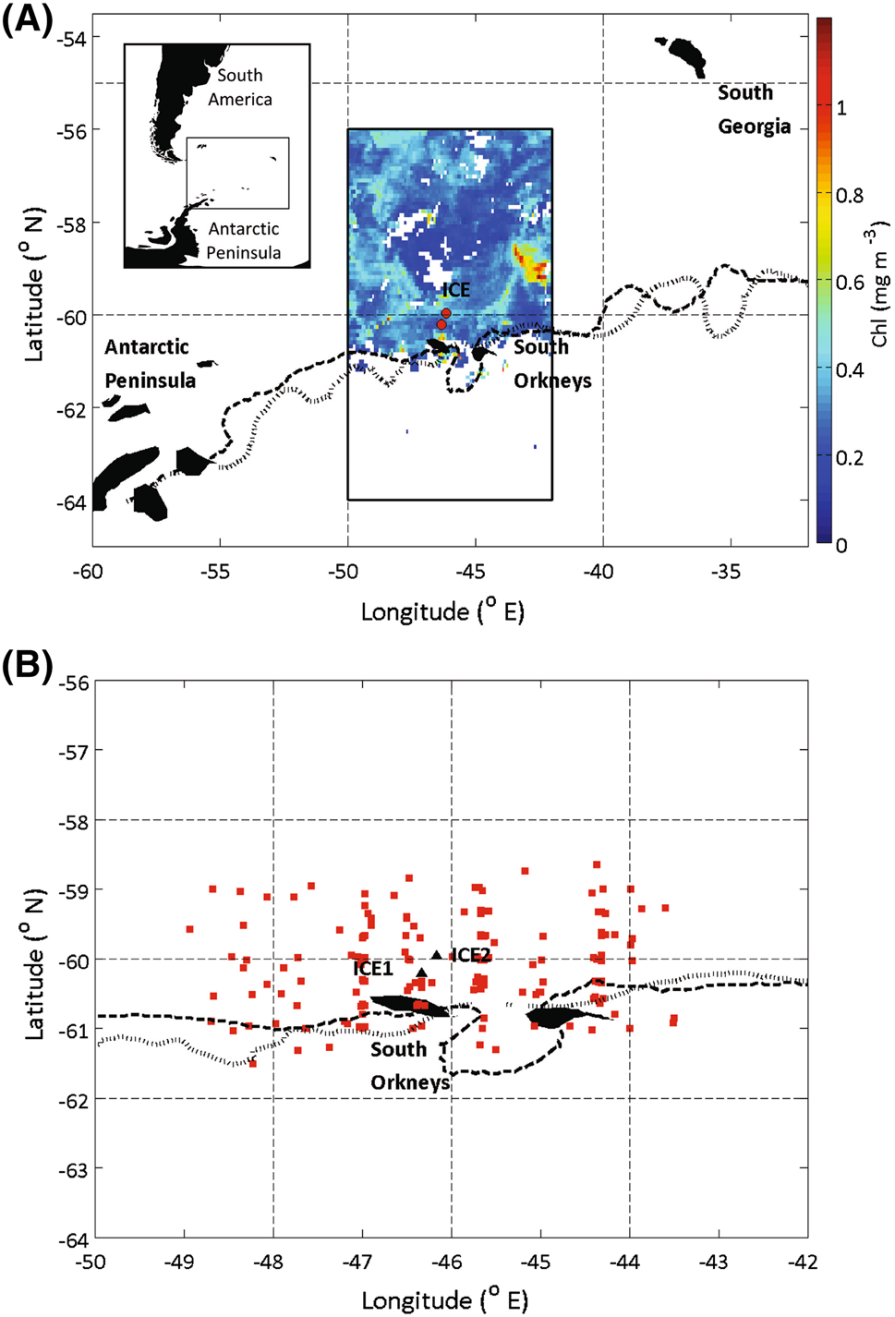
**a** ICE station locations (indicated by *red circles*) overlain on MODIS Aqua satellite chlorophyll (mg m^−3^) for December 2014 (chlorophyll shown for region covered by **b**). Top left inset shows larger regional scale for context. Position of ice edge on December 14th 2014 (*thick black dashed line*) and December 3rd 2013 (*black dotted line*) (OSTIA sea ice data). **b** Location of krill density samples taken from KRILLBASE for stations ICE1 and ICE2 (*red squares*). ICE1 and ICE2 stations are shown by *large black triangles*. (Color figure online)

Faecal pellet (FP) fluxes were estimated aboard RRS *James Clark Ross* during cruises JR291 and JR304 to the Scotia Sea, Antarctica in austral spring 2013 and 2014, respectively (Fig. 1a). Samples were obtained in the marginal ice zone near the South Orkney Islands at stations ICE1 (−60.21°N, −46.34°E) and ICE2 (−59.96°N, −46.16°E) during cruise JR291 (01/12/2013) and ICE2 only during cruise JR304 (26/11/2014). There was no ice cover at the time of sampling, but satellite data reveal intermittent ice cover in the weeks prior to sampling (see Figure S1).

### Particle collection

Sinking particles were collected using Marine Snow Catchers (MSCs). These are large (95 L) PVC closing water bottles designed to minimise turbulence (Riley et al. 2012; Cavan et al. 2015; Belcher et al. 2016a, b). Vertical profiles of temperature, salinity, and fluorescence were taken prior to MSC deployments using a conductivity–temperature–depth (CTD) unit (Seabird 9Plus with SBE32 carousel) to define the base of the mixed layer and thus MSC deployment depths. To assess the attenuation of FP in the upper mesopelagic, two MSCs were deployed in quick succession at 10 and 110 m below the base of the mixed layer depth (MLD), herein referred to as MLD +10 and MLD +110, respectively. MSCs were deployed during the day at ICE2 during both cruises and, due to logistical constraints, 1–1.5 h after sunset at ICE1.

Following deployment and recovery of the MSCs (which typically took 0.25–0.5 h), they were left on deck for a 2 h settling period, before draining the bottle and carefully removing the particle collector tray (divided into four quadrants for sample splitting) from the base which, was stored at 2–4 °C for further analysis. Fast-sinking particles are operationally defined here as particles sinking fast enough to reach the base of the MSC during this time (Riley et al. 2012), which, given the height of the snow catcher of 1.53 m, requires a minimum sinking rate of 18.4 m d^−1^ for particles to reach the base of the MSC. This settling period is more than sufficient to allow FP to reach the tray based on previous direct measurements of FP sinking velocity in the Southern Ocean which range from 27 to 1218 m d^− 1^ (Atkinson et al. 2012; Cavan et al. 2015; Belcher et al. 2016b).

### Particle type

During both JR304 and JR291, 1–4 sample splits were photographed using an Olympus SZX16 microscope with Canon EOS 60D camera and Olympus BX-SZX Micro Cam and particles classified into: krill FP, other FP, phytodetrital aggregates, phytoplankton cells, and other phytodetritus. Phytodetrital aggregates were identified as aggregations >0.1 mm equivalent spherical diameter (ESD) containing phytoplankton cells and other phytodetrital material. Individual particle dimensions were measured using ImageJ and volumes calculated using formulae for a sphere, ellipsoid, or cylinder depending on particle shape. *E. superba* FP were identified via comparison with freshly egested FP (Belcher et al. 2016b). FP carbon contents were calculated from measured volumes based on direct measurements at the study site (see in the following). Conversions from volume to carbon for phytoplankton cells were based on the equation of Menden-Deuer and Lessard (2000) for diatoms >3000 μm^3^: log (pgC_cell_^−1^) = *−*0.933 + 0.881 log[volume (μm^3^)], and conversions from phytodetrital aggregate and other phytodetrital material volume to carbon based on the equation of Alldredge (1998): μC_agg_^−1^ = 0.99 [volume (mm^3^)^0.52^]. The use of literature conversions introduces uncertainties into our results, but our use is consistent with previous studies (e.g., Alldredge 1998; Laurenceau-Cornec et al. 2015). Furthermore, we are more interested here in determining the relative reduction, rather than the absolute amount, of flux between depths, so reducing the effect of these uncertainties.

In this study, we focus on FP to assess the importance of krill FP to total POC flux. All FP were counted and their length and widths measured manually using ImageJ. Equivalent spherical diameters (ESD) were also calculated. Volumes were calculated from these measurements using the formula for a cylinder, and FP carbon content calculated according to an FP carbon to volume ratio of 0.032 mg C mm^−3^ (range 0.022–0.042 mg C mm^−3^) based on measurements made on FP (10–15 per replicate filter) collected from Bongo nets at ICE2 during JR304 (see Belcher et al. (2016b) for details of Bongo net deployments). FP were rinsed three times in filtered sea water, photographed under a microscope to obtain size measurements, filtered onto pre-combusted glass fibre filters (25 mm diameter GF/F, Whatman), and oven dried at 50 °C for analysis of POC. Filters were then fumed with 37% HCl in a vacuum desiccator for 24 h, and dried for 24 h at 50 °C, before placing both filters and filter blanks in pre-combusted (450 °C, 24 h) tin capsules as in Hilton et al. (1986), and measuring POC in a CE-440 Elemental analyser (Exeter Analytical.285 Inc).

### Faecal pellet flux

Following calculation of the total mass (*m*) of sinking FP carbon in the MSC, FP flux (*F*) was calculated as follows:

1 $$F\;\left( {{\text{mg}}\,{\text{~C~}}\,{{\text{m}}^{ - {\text{2}}}}\,{{\text{d}}^{ - {\text{1}}}}} \right)\;=~\;\frac{m}{A}\;\; \times \;\;\frac{w}{h}~,$$

where *A* refers to the area of the MSC opening based on inner MSC diameter, *w* the measured sinking velocity (m d^−1^) from laboratory measurements, and *h* the height of the snow catcher (1.53 m). Krill FP flux was calculated based on krill FP sinking velocities only.

During JR291, FP sinking velocities were measured in a temperature controlled laboratory (at 2 °C) using a 1 L graduated glass cylinder (7 cm diameter). Laboratory temperatures were at most ~3 °C warmer than in situ water temperatures which could result in overestimations of sinking speed of up to 15% based on theoretical calculations of the effects of viscosity (Taucher et al. 2014). However, observational studies have not observed differences in sinking velocity at different temperatures (Trull et al. 2008; Iversen and Ploug 2013), so this small difference in temperature is unlikely to bias our measurements. FP were carefully removed from the particle collector tray using a plastic pipette and transferred into a graduated cylinder which was filled with seawater collected from the MSC at the ICE station. The sinking velocity of each FP was calculated from the average of the time taken to sink past two marked distances (10 cm apart), with the starting point more than 10 cm from the water surface. Results were discarded where the walls of the cylinder were observed to interfere with the sinking FP. During JR304, sinking velocities were measured in a temperature controlled (at 4 °C) flow chamber system (Ploug and Jorgensen 1999) containing filtered sea water (0.22 µm filter) taken from the MSC deployed at ICE stations. FP were placed carefully in the chamber and three measurements of the sinking velocity made for each FP by suspending the FP with an upward flow (Ploug and Jorgensen 1999). Sinking velocity measurements were limited to those particles visible by eye (ESD >0.15 mm). Sinking velocities measured during JR291 and JR304 by these two different methods were not significantly different (Student’s *t* test, *p* = 0.2), suggesting that the use of two different methods to measure sinking velocity did not bias our results.

### Faecal pellet flux attenuation

The rate of mesopelagic FP flux attenuation was assessed by fitting a power-law function (Martin et al. 1987) to the FP flux data:

2 $${F_z}={F_{{z_0}}} \times {\left( {z/{z_0}} \right)^{ - b}},$$

where *z* is the depth of the flux (m), and *F*_*z0*_ is flux (mg C m^−2^ d^−1^) at the reference depth. A high absolute value of *b* corresponds to high attenuation (shallow remineralisation) and vice versa. We calculate attenuation rates through the mesopelagic (between MLD +10 and MLD +110) based on our MSC estimates of FP flux which we define as *b*_MSC_.

## Results

### Oceanographic setting

**Fig. 2:**
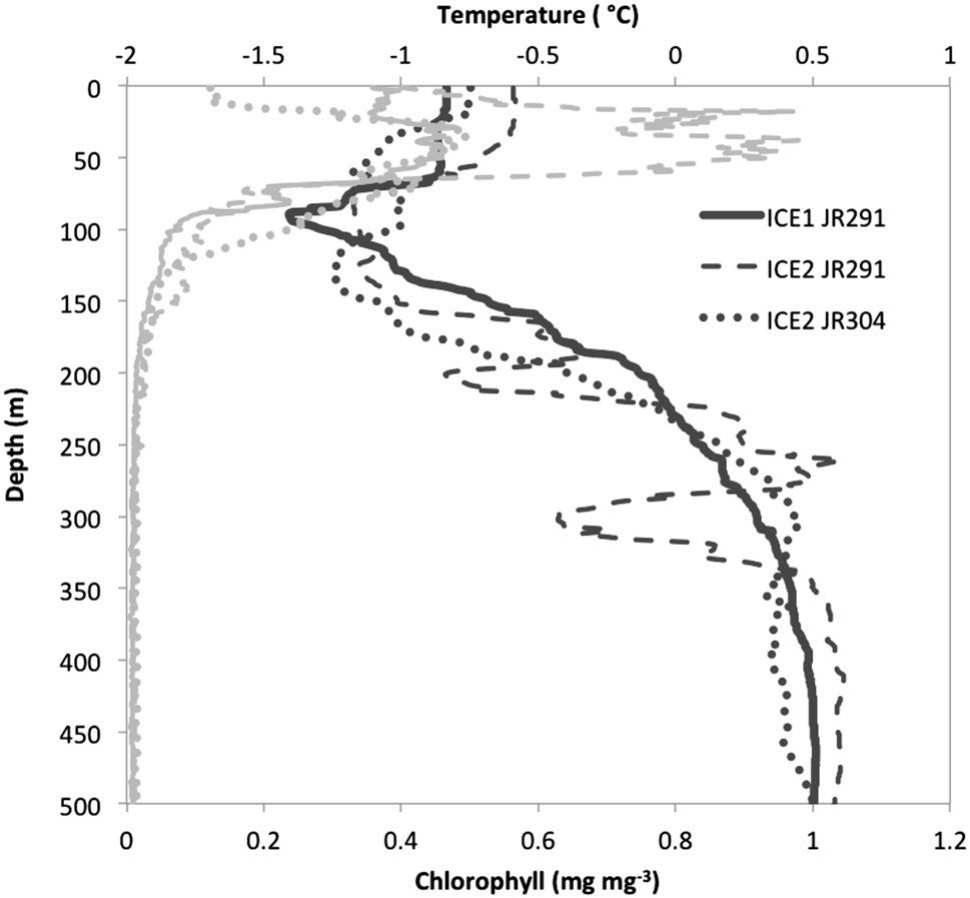
Vertical profiles of temperature (*dark grey lines*) and chlorophyll a (*light grey lines*) from CTD deployments at ICE1 (*solid line*), and ICE2 (*dashed line*), during cruises JR291, and ICE2 during JR304 (*dotted line*)

Surface temperatures were consistent between sites and between years ranging from −0.83 to −0.59 °C (Fig. 2). At ICE1 JR291 and ICE2 JR304, chlorophyll a peaked at 0.5 mg m^−3^ at 30 m. Chlorophyll a was elevated at ICE2 JR291, peaking at 1 mg m^−3^ at 38 m (Fig. 2).

### Particle flux and type

**Fig. 3:**
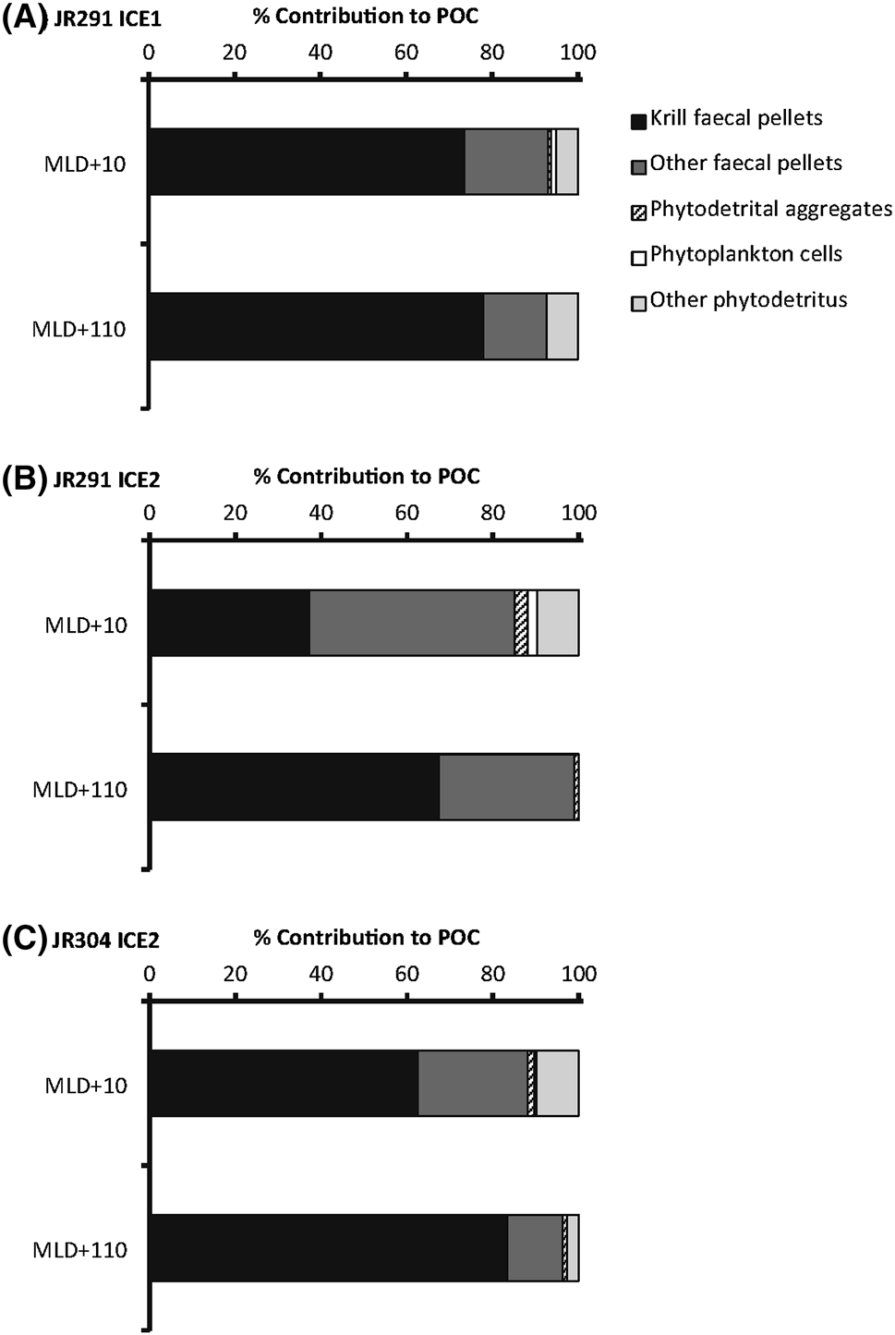
Type of fast-sinking particulate organic carbon at (**a**) ICE1 JR291, (**b**) ICE2 JR291, and (**c**) ICE2 JR304 stations based on microscope analysis and calculated carbon content. *Black* krill faecal pellets, dark *grey* other faecal pellets, *hashed* phytodetrital aggregates, *white* phytoplankton cells and *light grey* other phytodetritus

FP were the dominant component of the flux at all stations (Fig. 3), accounting for 85.0–93.1% of the sinking POC at MLD +10, and 93–99% at MLD +110. As we were only able to directly measure the POC content for FP, there is some uncertainty in these percentages; however, even in terms of numerical abundance, FP accounted for 52–58% of total sinking particle abundance at MLD +10 and 56–95% at MLD +110. Most of these FP belonged to Antarctic krill, with the exception of the MLD +10 sample at ICE2 JR291, where krill FP accounted for 43.6% of the FP POC. The remaining FP were smaller cylindrical pellets that may originate from copepods or smaller euphausiid species. Krill FP were on average 0.15, 0.13, and 0.14 mm in width at MLD +10, and 0.15, 0.17, and 0.16 mm at MLD +110 at ICE1 JR291, ICE2 JR291, and ICE2 JR304, respectively. Krill FP lengths were on average 1.03, 0.70, and 1.28 mm at MLD +10, and 0.69, 0.91, and 0.90 mm at MLD +110 at ICE1 JR291, ICE2 JR291, and ICE2 JR304, respectively. However, as krill FP are produced in strings which can be easily broken, we calculate FP fluxes in terms of carbon rather than absolute abundance.

**Fig. 4:**
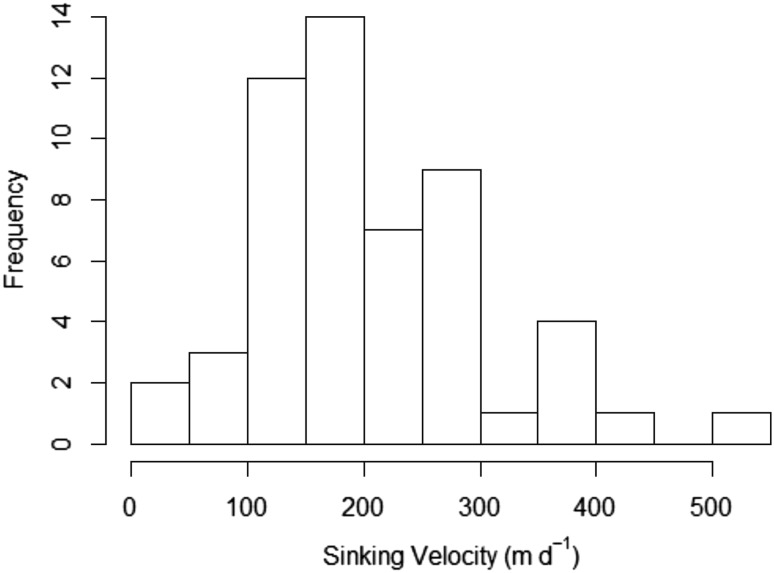
Distribution of krill faecal pellet sinking velocities (m d^−1^) measured at stations ICE1 and ICE2 during JR291 and JR304

**Fig. 5:**
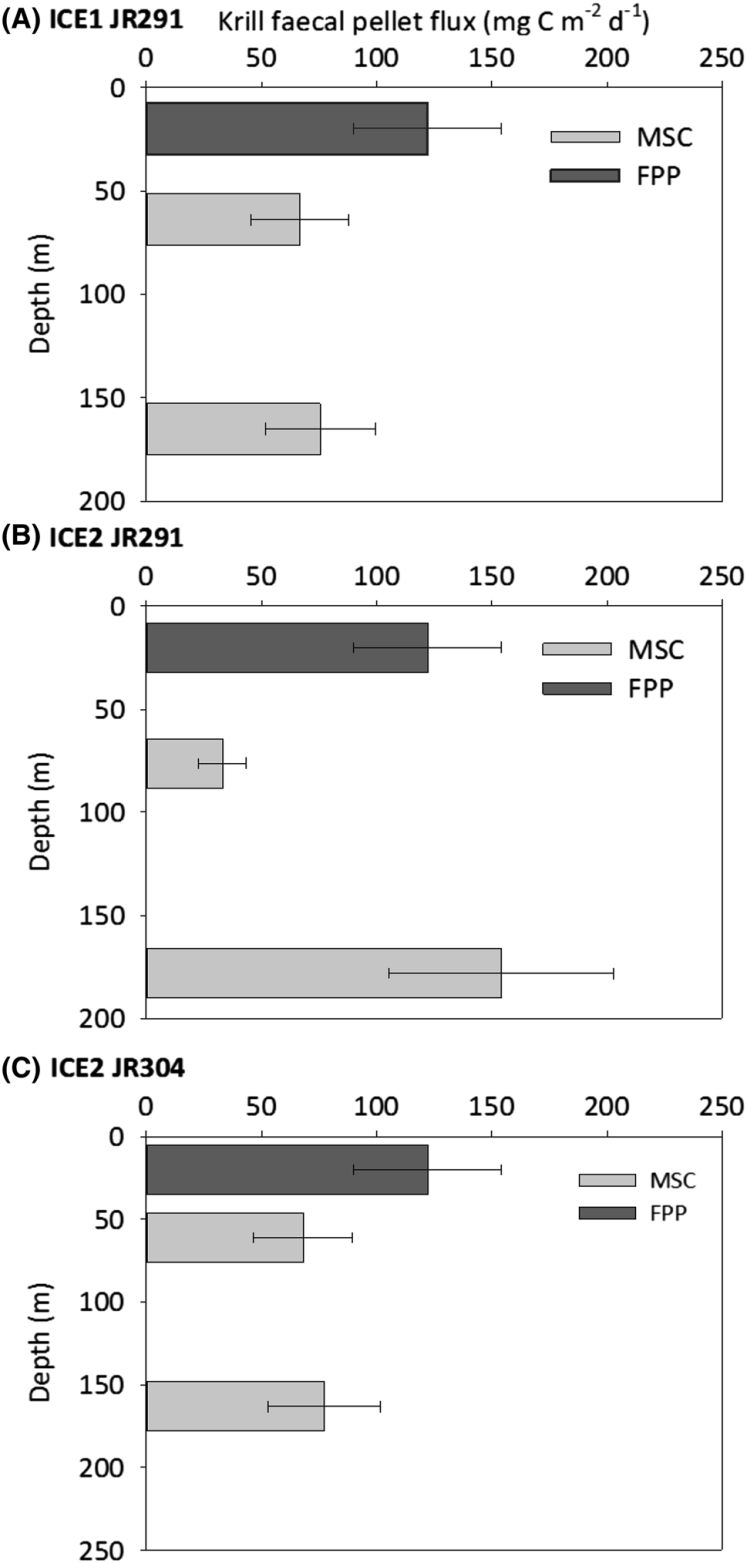
Krill faecal pellet (FP) fluxes estimated in the South Orkneys at (**a**) ICE1 JR291, (**b**) ICE2 JR291, and (**c**) ICE2 JR304. Krill FP fluxes estimated in Marine Snow Catchers (MSC) at the mixed layer depth +10 m and the mixed layer depth +110 m are shown by *light grey bars* (*error bars* show maximum and minimum fluxes based on the range of measured FP particulate organic carbon contents). Predicted krill FP production (FPP) at a depth of 20 m is shown by *dark grey bars* based on an egestion rate of 3.2 mg C ind^−1^ d^−1^ from Clarke et al. (1988), with *error bars* showing maximum and minimum fluxes based on krill densities from KRILLBASE (mean ± 1SE)

Median krill FP sinking velocities were 172, 267, and 161 m d^−1^ at ICE1 JR291, ICE2 JR291, and ICE2 JR304, respectively, ranging from 15 to 507 m d^−1^ (*n* = 54, Fig. 4). A Student’s *t* test on sinking velocities at each site revealed no statistically significant differences between means (*p* > 0.05). Krill FP fluxes (Fig. 5) were very similar at ICE1 JR291 and ICE2 JR304 at both MLD +10 (66.7 and 68.0 mg C m^−2^ d^− 1^) and MLD +110 (75.5 and 77.3 mg C m^−2^ d^−1^), showing a slight increase in flux with depth (Table 1). Conversely, at ICE2 JR291, a large increase in krill FP flux was observed between MLD +10 and MLD +110 (33.0 to 154.1 mg C m^−2^ d^−1^).

**Table 1:** Marine Snow Catcher deployment table for cruises JR291 and JR304 to the South Orkneys, Antarctica, showing total faecal pellet (FP) and krill FP carbon fluxes

Site	Date	Time (GMT)	Depth (m)	FP flux (mg C m^−2^ d^−1^)	Krill FP Flux (mg C m^−2^ d^−1^)
ICE1 JR291	01/12/2013	01:45	64	88.5	66.7
		02:21	165	94.3	75.5
ICE2 JR291	01/12/2013	12:58	76	74.2	33.0
		13:37	178	221.4	154.1
ICE2 JR304	26/11/2014	16:44	61	92.9	68.0
		17:12	163	86.1	77.3

### Faecal pellet flux attenuation

At all sites, we observed an increase in krill FP between MLD + 10 and MLD +110 in MSC samples resulting in negative attenuation coefficients (*b*_MSC_) of −0.13, −1.81, and −0.13 at ICE1 JR291, ICE2 JR291, and ICE2 JR304, respectively.

## Discussion

### Faecal pellet flux

Krill FP were the dominant component of the POC flux at ICE1 and ICE2 stations (Fig. 3) highlighting their importance for the transfer of POC through the upper mesopelagic layer in this MIZ region. These high fluxes may, at least in part, be driven by the high FP sinking velocities that we measured. Our MSC estimated krill FP fluxes at ICE1 and ICE2 (75.5–154.1 mg C m^−2^ d^−1^) are high compared to fluxes of cylindrical FP (presumed from krill) measured in sediment traps deployed on the Western Antarctic Peninsula at 170 m in summer (11.36 mg C m^−2^ d^−1^) (Gleiber et al. 2012). However, Gleiber et al. (2012) also recorded peaks in cylindrical krill FP flux of up to 125.5 mg C m^−2^ d^−1^ in summer. Krill FP fluxes compare quite well to measurements made at 50 m in the MIZ of the Scotia Sea [FP fluxes of ~60 mg C m^−2^ d^−1^, dominated by krill (Cavan et al. 2015)]. Our study, therefore, agrees with previous studies that Antarctic krill FP can make a major contribution to POC fluxes in the upper mesopelagic in the MIZ in spring and summer (75–100%, Gleiber et al. 2012; Cavan et al. 2015). We, therefore, suggest that areas of high krill density warrant more detailed research to assess their contribution to bathypelagic POC fluxes and long-term carbon sequestration.

### Faecal pellet production and attenuation

We observed an increase in krill FP flux between MLD +10 and MLD +110 (negative attenuation) at all sites suggesting non-steady-state FP production, minimal attenuation with depth, FP production between the measurement depths, and/or inputs via lateral advection. Satellite-derived surface current data (OSCAR, 1/3 degree grid, 5 day resolution) suggest that surface velocities were <0.15 m s^−1^ at the ICE stations during our study period, and thus, lateral advection was likely small.

At ICE1 JR291 and ICE2 JR304, only a slight increase in krill FP with depth was observed (*b*_MSC_ = −0.13), which we suggest is a result of low attenuation and an additional input via satiation sinking (i.e., sinking as a result of being full), as supported by previous studies in the region (Lancraft et al. 1991; Tarling and Johnson 2001). Tarling and Johnson (2001) estimate that krill spent 40% of the night-time period below the surface mixed layer due to satiation sinking, and egest 1.7 mg C ind. ^−1^ d^−1^ during this time. This would equate to 81.6 mg C m^−2^ d^− 1^ for a typical mean krill density of 38 ind. m^−2^. Tarling and Johnson (2001) estimate that krill can descend 9–46 m during satiation sinking, and with most krill in the region occurring within the upper 50 m in spring (Fielding et al. 2012), this could account for our observed increase in FP flux between sampling depths.

FP production at depth due to satiation sinking cannot, however, explain the observed fourfold increase in krill FP between MLD +10 and MLD +110 at ICE2 JR291, even in the unlikely situation that no remineralisation of the FP produced during satiation sinking occurred. We hypothesise that FP production between MLD +10 and MLD +110 due to diel vertical migration (DVM), short-term intermittent vertical migrations (Godlewska and Klusek 1987) or simply a deeper distribution of krill, could account for increases in FP abundance with depth at ICE2 JR291. ICE2 JR291 was sampled during the day (10:58–11:37 local time), and if gut passage times were sufficient, then DVM may explain the high FP fluxes at this site. However, ICE2 JR304 was also sampled during the day (14:44–15:12 local time), and similarly, large increases in FP fluxes with depth were not observed. Most krill swarms in the region of our study site occur at depths <60 m in spring (Fielding et al. 2012) and in late spring–early summer DVM of krill are small in the South Orkney Islands area (Taki et al. 2005). We, therefore, consider it unlikely that DVM alone could result in the large increase in FP at depth that we observed at 178 m at ICE2 JR291.

Considering the lower percentage contribution of krill FP at MLD +10 at this site compared to ICE1 and ICE2 JR304 (43.6% compared to >85%, Fig. 3), this apparent increase with depth may reflect non-steady-state FP production, with increased production (perhaps associated with the increased chlorophyll concentration (Fig. 2), or with the passing of a krill swarm) prior to sampling resulting in higher FP fluxes at MLD +110 as the FP sank through the ocean. Although dedicated acoustic transects were not carried out during our study preventing the calculation of krill densities at the time of sampling, the shipboard EK60 120 kHz echosounder highlights that a number of large krill swarms (two orders of magnitude larger in area than observed at our other study sites) were present, at mean depths of <50 m, up to 5 h prior to sampling at ICE2 JR291. Acoustic observations, therefore, support our hypothesis that the passing of krill swarms can drive higher fluxes of FP through the mesopelagic as also found in other regions (Cadée 1992).

The high fluxes of krill FP measured at both MLD +10 and MLD +110 at all stations (Table 1) could be due to high FP production (FPP) at the depth of krill feeding and/or due to low attenuation. We have made preliminary estimates of krill FPP based on krill densities obtained from the literature (Atkinson et al. 2008; Fielding et al. 2012; Skaret et al. 2015; Table 2), and krill FP egestion rates measured by Clarke et al. (1988). Clarke et al. (1988) carried out FP egestion experiments on *E. superba* collected from Arthur Harbour, on Anvers Island, Antarctica, as well as the Bransfield Strait, Antarctica. They measured a range in egestion rates (0.25–2.35 mg FP dry weight ind^−1^ h^−1^), giving an egestion of 0.7–6.3 mg C ind^−1^ d^−1^ assuming an organic C content of 11.15% dry weight [average of open ocean sites (8.1–13.7%) from Clarke et al. (1988)]. We take their ‘representative’ egestion rate of 1.2 mg FP dry weight ind^−1^ h^−1^, giving a rate of 3.2 mg C ind^−1^ d^−1^. The range in literature estimates of krill density is high, 28–2009 ind. m^− 3^ (Table 2), highlighting the patchy nature of krill swarms. The bulk of estimates lies between 28 and 700 ind. m^−3^ (Atkinson et al. 2008; Fielding et al. 2012), with one high record of 1128 ind. m^−3^ from the KRILLBASE database of Atkinson et al. (2008) and the high value of 2009 ind. m^−3^ from the acoustic data of Skaret et al. (2015). Applying an egestion rate of 3.2 mg C ind^−1^ d^−1^ to krill densities of 28–700 ind. m^−3^ gives FPP of 90–2248 mg C m^−2^ d^−1^, highlighting that FPP could indeed be very high. When high krill densities occur, this could drive much higher FP fluxes to the mesopelagic without the need for low rates of attenuation, a scenario which may have been captured by our sampling at ICE2 JR291 (Fig. 5b). The apparent negative attenuation rate (i.e., an increase in FP flux with depth) calculated at this station may, therefore, be explained by the episodic passing of a krill swarm prior to the time of sampling (supported by the semi-quantitative shipboard acoustic observations described above). This site may, therefore, not represent steady-state conditions, reducing the validity of estimates of attenuation at this site.

**Table 2:** Estimates of krill density in the study region of the South Orkneys based on previous studies

Data Type	Source	Region	Year	Season	Mean krill density (ind. m^−2^)^a^
Net (KRILLBASE)^c^	Atkinson et al. (2008)	300 × 300 km box centred on ICE1	1926–2004	Standardised to January	38 (±10, 0–1128)^b^
Acoustic^d^	Fielding et al. (2012)	South Orkneys: their Sp3	2006	November	700
			2008	January	122
		South Orkneys: their Sp4	2006	November	28
			2008	January	36
Acoustic^e^	Resampled from Skaret et al. (2015)	25 km to the north and south of ICE1	2014	January	2009

^a^Acoustic estimates have been converted using size–weight conversions (Kils 1981)

^b^Mean (±standard error, range)

^c^A historical database (KRILLBASE) of 9922 individual net hauls taken in the Southern Ocean since 1926

^d^50 km surveys carried out close to the ICE stations by the RRS *James Clark Ross* using a Simrad EK600 echosounder (38, 120, 200 kHz)

^e^Surveys were carried out by the FV *Saga Sea* in the region of the South Orkneys using a 38 and 120 kHz echo sounder; we resampled the transect line nearest to our study site (transect following − 46.5°E), analysing sample bins with the range of 25 km to the north and south of the ICE1 station

Based on the above calculation of FPP, we estimate the possible ‘net’ attenuation between FP production at a depth of 20 m [based on mean krill swarm depths of 18.9 m measured in the southern Scotia Sea in spring (Fielding et al. 2012)] and our measurements of krill FP flux at MLD +110 at ICE1 JR291 and ICE2 JR304, which we define here as *b*_FPP_. We do not include the FP flux measured at MLD +110 during ICE2 JR291 in our calculations of *b*_FPP_ to avoid bias due to potential non-steady-state conditions. The calculated range in *b*_FPP_ of 0.07–1.61 highlights that, depending on the krill density, it is possible that net attenuation rates could be very low, but there could also be scenarios, where attenuation rates are high.

### Sensitivity analysis

Considering the range in possible krill densities and egestion rates, we conduct a sensitivity analysis to assess the validity of our estimates of krill density, FPP, and b_FPP_. Accurate measurements of krill FP egestion rates are not easy to obtain due to the complex behaviour of krill and the difficulty in replicating natural conditions (Schmidt and Atkinson 2016; Tarling and Fielding 2016). We did not directly measure krill FP egestion rates which increase the uncertainty in our calculated *b*_FPP_. We conduct a sensitivity analysis (Table 3) using the range of FP egestion rates measured by Clarke et al. (1988) (0.7–6.3 mg C ind^−1^ d^−1^), which also encompasses other krill FP egestion estimates from the literature [0.75–2.5 mg C ind^−1^ d^−1^, Nordhausen and Huntley (1990), Pakhomov et al. (1997), Atkinson et al. (2012)]. This results in *b*_FPP_ of between −0.73 and 0.87 for minimum FPP rates, and between 0.33 and 1.92 for maximum FPP rates based on mean KRILLBASE krill densities and the acoustic estimates of Fielding et al. (2012). However, using our highest krill density estimate from Skaret et al. (2015), we calculate attenuation rates of 1.36 and 2.43 based on minimum and maximum FPP, respectively. The density of krill is, therefore, the largest uncertainty in our estimates of FPP and b_FPP_.

**Table 3:** Sensitivity analysis of attenuation rates (b_FPP_) at ICE1 JR291 and ICE2 JR304

Site	Krill abundance (ind. m^−2^)	Krill egestion rate (mg C ind.^−1^ d^−1^)	Krill FP carbon content (% of DW)	Attenuation rate (*b*_FPP_) ^d^
ICE1 JR291	28–700 (2009)	0.67	11.15^a^	−0.73–0.87 (1.36)
	28–700 (2009)	6.29	11.15^a^	0.33–1.92 (2.43)
	28–700 (2009)	0.23^b^	3.8	−1.17–0.36 (0.86)
	28–700 (2009)	9.64^c^	17.1	0.60–2.13 (2.63)
ICE2 JR304	28–700 (2009)	0.67	11.15^a^	−0.75–0.86 (1.36)
	28–700 (2009)	6.29	11.15^a^	0.32–1.93 (2.43)
	28–700 (2009)	0.23^b^	3.8	−1.18–0.35 (0.85)
	28–700 (2009)	9.64^c^	17.1	0.60–2.13 (2.63)

Attenuation rates have been calculated based on the range of krill abundances (ind. m^−2^) from KRILLBASE (mean ± 1SE) and acoustic estimates from Fielding et al. (2012), the range of faecal pellet egestion rates (mg C ind^−1^ d^−1^) from Clarke et al. (1988), and krill faecal pellet (FP) carbon contents from Clarke et al. (1988) and Atkinson et al. (2012). We also give attenuation rates based on the very high krill density estimate from summer 2014 krill density data (Skaret et al. 2015), shown in *brackets*

^a^Average of open ocean sites (8.1–13.7%) from Clarke et al. (1988)

^b^Based on minimum egestion rates (Clarke et al. 1988) and minimum FP carbon contents measured by Atkinson et al. (2012) in the Scotia Sea in spring

^c^Based on maximum egestion rates (Clarke et al. 1988) and maximum FP carbon contents measured by Atkinson et al. (2012) in the Scotia Sea in spring

^d^Attenuation rates calculated between our predicted FPP at a depth of 20 m [based on mean krill swarm depths of 18.9 m measured in the southern Scotia Sea in spring (Fielding et al. 2012)] and our estimated FP fluxes at the mixed layer depth +110 m. We exclude the very high flux value of ICE2 JR291 (likely due to non-steady-state conditions and the passing of a krill swarm, see “Discussion”)

Clarke et al. (1988) measured a large variation in the rate of total FP egestion, but found that the loss of organic material showed no significant daily variation during experiments in summer, increasing our confidence in the value for FP carbon content used here. However, Manno et al. (2015) observed changes in FP carbon content seasonally and Atkinson et al. (2012) found that egestion rates and absorption efficiencies varied with diet and food availability, measuring carbon contents of 3.8–17.1% of the FP dry mass in the Scotia Sea in spring. Using this range in FP carbon content and the aforementioned range in FP egestion rates, we calculate FP carbon egestion rates ranging from 0.23 to 9.64 mg C m^−2^ d^−1^. Again, this produces a wide range in attenuation rates (Table 3), highlighting the need for more constrained measurements of krill density and krill FP production alongside krill FP measurements at depth to determine just how low these attenuation rates are.

### Krill FP attenuation in the Southern Ocean

Although high fluxes of krill FP have been noted elsewhere before, these have often only been measured at one depth so prohibiting quantification of attenuation rate (Dunbar 1984; Bathmann et al. 1991; Gleiber et al. 2012), or visual observations of krill FP have been made rather than measuring FP carbon fluxes directly (Bodungen et al. 1987; Wefer et al. 1988), or attenuation rates have not been calculated (Cadée et al. 1992; Accornero et al. 2003). The large seasonal and latitudinal variability in particle flux in the Southern Ocean (e.g., Honjo et al. 2000, 2008; Dubischar and Bathmann 2002 and refs within) makes it difficult to compare studies and combine all data to create a Martin type curve for sinking krill FP in the Southern Ocean. Where possible, we calculate FP attenuation rates from the literature to compare to our estimates of krill FP attenuation rate (Table 4). Previous studies, as collated in Table 4 (Wefer et al. 1988; Cadée et al. 1992; González 1992; Accornero et al. 2003; Cavan et al. 2015), highlight just how variable krill FP flux attenuation rates can be (−1.81 to 2.46).

**Table 4:** Comparison of krill faecal pellet (FP) fluxes and attenuation rates

Source	Region	Depth (m)	Season	Krill FP flux (mg C m^− 2^ d^− 1^, or nFP m^− 2^ d^− 1^)^a^	Attenuation rate (*b* value)	Notes
This study	South Orkneys	64	December	66.7	−0.13	ICE1 JR291
		165		75.5		
		76	December	33.0	−1.81	ICE2 JR291
		178		154.1		
		61	November	68.0	−0.13	ICE2 JR304
		163		77.3		
Cadée et al. (1992)	Scotia-Weddell seas	50	December	166	1.19	Smaller FP at 50 m
		150		45		
		75	December	220	0.10	Krill swarm observed
		150		205		
		75	December	122	0.92	
		150		64.5		
Wefer et al. (1988)^b^	Bransfield Strait	494	January	281.2	0.60	Productive period, krill FP dominant
		1588		139.9		
Accornero et al. (2003)	Ross sea	180	April		0.62	Cylindrical faecal fragments —believed to be krill or large copepods
		868				
		180	Annual mean	0.05	0.32	
		868		0.03		
Cavan et al. (2015)^c^	Scotia Sea	70	January	58.6	−0.32	Marginal ice zone station 12, krill FP dominant
		170		77.9		
González (1992)^d^	Scotia-Weddell seas	50	December–January	10	0.63	Transect (I) 49 **°**W
		150		5		
		50	December–January	5.5	2.18	Transect (II) 47 **°**W
		150		0.5		
		50	December–January	3	−1.14	Transect (III) 49 **°**W
		150		10.5	0.22	
		300		9		
		50	December–January	22.5	2.46	Station 157
		150		1.5		

^a^All fluxes refer to the carbon flux, with the exception of Cadée et al. (1992), where fluxes are given in terms of the number of krill FP strings

^b^Fluxes are for total particulate organic carbon

^c^Fluxes are for all FP, but krill FP were dominant

^d^Fluxes are FP in terms of FP dry weight, and have been estimated from Figs. 3 and 5 of González (1992)

Variability in attenuation rates may relate in part to the patchiness of krill swarms but also to the community composition in terms of the abundance of smaller flux feeding species. Previous studies have suggested that the density of individuals in krill swarms likely results in a ‘rain’ of FP which may overload detrital feeders and pass mostly undisturbed through the upper mesopelagic (Atkinson et al. 2012; Belcher et al. 2016b; Barnes and Tarling, in press). This has been hypothesised as an explanation for high numbers of krill pellets collected in sediment traps in the meso- and upper bathypelagic; however, full understanding of the processes driving these fluxes is still lacking. Lower abundances of small copepods such as *Triconia* spp. (previously *Oncaea* spp.) and *Oithona* spp. (often associated with flux feeding, fragmentation, and increased recycling) at our study site compared to more northerly sites in the Scotia Sea (Ward et al. 2012) may contribute to the low attenuation in FP flux (*b*_MSC_) that we observed (Belcher et al. 2016b). Higher abundances of flux feeding zooplankton can lead to increased retention of FP (González 1992); however, with the high FP production associated with high krill densities in krill swarms, this flux feeding capacity is still likely overloaded. Thus, it is likely that given high enough densities, krill can drive high FP fluxes through the mesopelagic even in the presence of this retention filter. The zooplankton abundance and community structure is, therefore, an important control on flux attenuation (Belcher et al. 2016b).

We show that krill FP can make up a large component of the carbon flux in the South Orkneys MIZ region in spring, which is likely a combination of high FPP and low attenuation. Our MSC data, therefore, add to the evidence for high sinking rates of krill FP, high fluxes, and seemingly low attenuation rates. However, further studies are required to resolve respective contributions of FPP and attenuation to the net flux of krill FP to understand the mechanisms driving high krill FP fluxes in the mesopelagic. Consensus is needed on krill FP attenuation before we can start to model the role of krill in biogeochemical cycling. We suggest that calculating krill FP attenuation rates from measurements of krill density, FPP, and mesopelagic krill FP fluxes in other high-density krill areas in the Southern Ocean would enable the contribution of krill FP to the BCP to be more fully assessed, particularly with regard to whether it holds the same importance as in the present study region. This would require a series of acoustic transects for krill density and measurement of particle flux at regular spatial intervals within this survey grid. Direct measurements of krill FP egestion would also be needed, including measurement of FP carbon contents. The calculated krill FP attenuation rates would also allow predictions of how future changes in krill densities might impact the Southern Ocean BCP.

## Electronic supplementary material

Below is the link to the electronic supplementary material.

Supplementary material 1 (PDF 73 KB)
