# Supplementary material for: The potential role of Antarctic krill faecal pellets in efficient carbon export at the marginal ice zone of the South Orkney Islands in spring
Source: Polar Biol. 2017 Apr 13;40(10):2001–13. doi: 10.1007/s00300-017-2118-z (PMC6961482; doi:10.1007/s00300-017-2118-z)
Supplement: Supplementary file 1 — Supplementary material 1 (PDF 73 KB) [file 300_2017_2118_MOESM1_ESM.pdf]

Supplementary Material

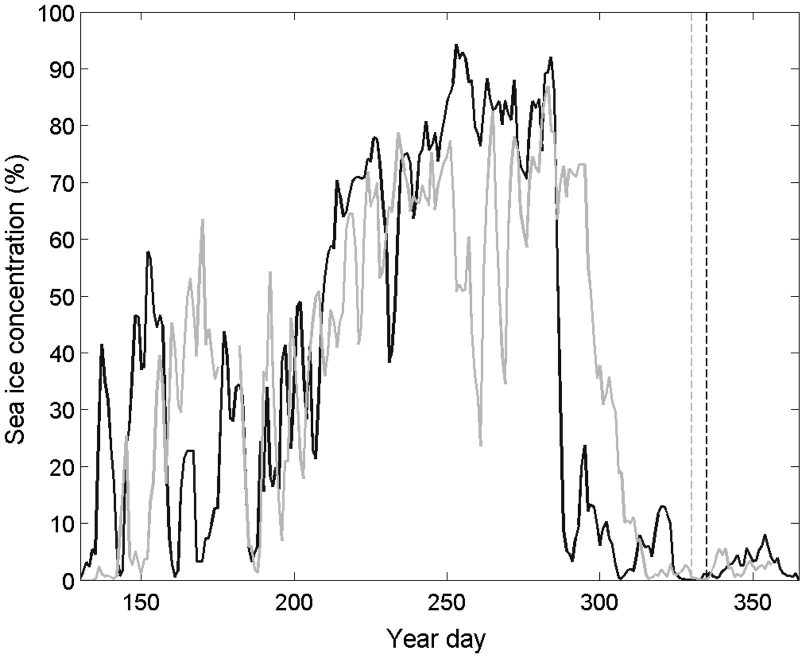

Fig S1 Sea ice concentrations (%) (OSTIA sea ice data, averaged over 1° box centred on ICE1) during 2013 (black solid line) and 2014 (grey solid line). Sampling dates for JR291 (black) and JR304 (grey) are shown by vertical dotted lines
